# Optical Imaging Approaches to Investigating Radiation Resistance

**DOI:** 10.3389/fonc.2019.01152

**Published:** 2019-11-05

**Authors:** Sina Dadgar, Narasimhan Rajaram

**Affiliations:** Department of Biomedical Engineering, University of Arkansas, Fayetteville, AR, United States

**Keywords:** raman spectroscopy, diffuse reflectance spectroscopy, optical metabolic imaging, nonlinear optical microscopy, mitochondrial organization, radiation resistance

## Abstract

Radiation therapy is frequently the first line of treatment for over 50% of cancer patients. While great advances have been made in improving treatment response rates and reducing damage to normal tissue, radiation resistance remains a persistent clinical problem. While hypoxia or a lack of tumor oxygenation has long been considered a key factor in causing treatment failure, recent evidence points to metabolic reprogramming under well-oxygenated conditions as a potential route to promoting radiation resistance. In this review, we present recent studies from our lab and others that use high-resolution optical imaging as well as clinical translational optical spectroscopy to shine light on the biological basis of radiation resistance. Two-photon microscopy of endogenous cellular metabolism has identified key changes in both mitochondrial structure and function that are specific to radiation-resistant cells and help promote cell survival in response to radiation. Optical spectroscopic approaches, such as diffuse reflectance and Raman spectroscopy have demonstrated functional and molecular differences between radiation-resistant and sensitive tumors in response to radiation. These studies have uncovered key changes in metabolic pathways and present a viable route to clinical translation of optical technologies to determine radiation resistance at a very early stage in the clinic.

## Introduction

About half of cancer patients from all cancer types are treated with radiation therapy either followed by or concurrently with surgery, chemotherapy, or other forms (1). However, despite the recent advances in targeted radiation therapy, several patients subsequently experience loco-regional recurrence due to acquired or intrinsic radiation resistance. The current standard of care to determine radiation response is an anatomical assessment of tumor volume shrinkage. This evaluation is typically performed 6–8 weeks after completion of treatment using X-ray Computed Tomography (CT) or Magnetic Resonance Imaging (MRI). There are currently no methods to determine radiation response either during or immediately after treatment. An early determination of radiation resistance could help physicians modify the radiation dosage to improve response rates and hence quality of life. The development of methods to identify radiation-resistant tumors early requires a better understanding of the biological mechanisms promoting radiation resistance.

Ionizing radiation functions by producing free radicals in cancer cells either directly in the DNA or indirectly in other molecules, primarily water (H_2_O). These radiation-induced free radicals, in the presence of O_2_, can generate peroxy radicals (DNA-OO·) capable of breaking chemical bonds and initiating a series of events which lead to DNA modification, and cell death (damage fixation). In contrast, lack of O_2_ leads to the reduction of free radicals in DNA and restoration of the original form of DNA (DNA-H) leading to cancer cell survival (2–4). Landmark studies in clinical head and neck cancer and soft-tissue sarcoma found that pre-treatment oxygenation levels were predictive of treatment response and disease-free survival (5–7). This important role of oxygen is the rationale for fractionated radiation therapy (2 Gy/day for 6–7 weeks), which is believed to re-oxygenate and radio-sensitize former hypoxic cells and hence, cause cell death via damage fixation (8–10). However, recent work has started to uncover a possible role for radiation-induced reoxygenation in also promoting radiation resistance through hypoxia-inducible factor (HIF).

Hypoxia leads to stabilization of HIF-1 (11). While HIF-1 expression is inhibited under oxygenated conditions via prolyl hydroxylases (PHDs), its transcription is significantly upregulated under hypoxic conditions (3, 12, 13). However, radiation-induced tumor reoxygenation can lead to activation of HIF-1 as well-through accumulation of reactive oxygen species (ROS), which is necessary and sufficient to stabilize HIF-1 (14). Nuclear accumulation of HIF-1 in response to ROS has been shown to promote endothelial cell survival and hence promote radiation resistance (15, 16). In a tumor bearing window chamber model, Moeller et al. demonstrated an increase in ROS during radiation-induced reoxygenation. Additionally, they showed that injecting hydrogen peroxide (H_2_O_2_) into the window chamber lead to an increase in HIF-1 expression (15). HIF-1 directly targets several glycolytic genes and leads to increased glucose catabolism under oxygenated conditions (17–20). HIF-1 trans-activates pyruvate dehydrogenase kinase (PDK), which inhibits pyruvate dehydrogenase and shunts pyruvate away from the mitochondria resulting in glucose catabolism to lactate even under oxygenated conditions (17, 18). Inhibition of HIF-1 and subsequent inhibition of PDK-1 restores glucose flux toward mitochondria and increases O_2_ consumption (21). Other studies have shown that HIF-1 and pyruvate kinase M2 exist in a positive feedback loop that enhances glycolysis under aerobic conditions (19, 20).

Zhong et al. demonstrated that scavenging ROS resulted in a reduction in post-radiation aerobic glycolysis without reducing the magnitude of reoxygenation (22).

The switch to increased glucose catabolism can promote radiation resistance through utilization of the pentose phosphate shunt (PPP) to maintain the NADPH-glutathione buffer and hence scavenge radiation-induced ROS. Inhibition of glucose flux through the PPP in combination with 2 Gy of radiation treatment significantly decreased cancer cell proliferation, especially in radiation-resistant cells (23). Increased glucose catabolism can also lead to increased production of lactate, an important ROS scavenger, leading to decreased radiation sensitivity (24, 25). Thus, in addition to being key hallmarks in the development of cancer, tumor oxygenation (or hypoxia) and metabolism play a significant role in the development of radiation resistance. Technologies that are sensitive to these key hallmarks and that can measure them both at the “bench” and “bedside” can provide powerful tools to shed light on radiation resistance.

Optical imaging can provide non-destructive and quantitative methods to reveal morphological and biochemical changes within cells and tissue across length scales in response to radiation therapy. Due to its non-destructive nature, optical imaging can be used to longitudinally monitor dynamic biological changes with high resolution to investigate the underlying mechanisms that promote radiation resistance. Given the low cost and non-ionizing nature of the light used, optical techniques are also well-positioned for clinical translation, especially for accessible tumors of breast, skin, oral cavity, and uterine-cervix. In addition, same instrumentation and quantitative models are frequently used to extract meaningful information from pre-clinical animal models. This review highlights recent work that used non-linear optical microscopy and diffuse optical spectroscopy to shed light on differences between radiation-resistant and sensitive cancer cells. Specifically, we highlight studies that identified differences in oxygenation or reoxygenation trends post-radiation therapy as well as those that investigate metabolic and molecular changes in the post-radiation tumor milieu. These studies encompass models ranging from *in vitro* cell culture to *in vivo* animal studies and indicate the great potential of optical imaging in the sphere of biological investigations of radiation resistance and the development of clinically translational optical technologies to benefit patients receiving radiation therapy.

## Optical Microscopy

Non-linear microscopy approaches, such as two-photon microscopy present numerous advantages over conventional single-photon microscopy (26). Because autofluorescence is generated through simultaneous absorption of two photons, the excitation wavelengths used are at twice the single-photon excitation wavelength and half the energy. Doubling the single-photon excitation wavelength usually places the non-linear excitation wavelength in the near-infrared range, which allows light to penetrate deeper within tissue (27). Additionally, the localization of autofluorescence to just the focal point of the objective provides an efficient method for rejecting out-of-focus light and minimizing photodamage to the sample. In this review, we discuss how two-photon excited fluorescence (TPEF) from two key metabolic cofactors—nicotinamide and flavin adenine dinucleotides (NADH and FAD, respectively), can provide a non-destructive metabolic profile of cells and how these approaches have been utilized to study the metabolic response to therapy in radiation-resistant and sensitive cancer cells.

### Cellular Metabolism

Non-linear optical microscopy is well-suited to provide non-invasive high-resolution 3D images of mitochondrial structure and function within live cells, tissues, and animals (27, 28). Through two-photon excited fluorescence (TPEF), the intrinsic fluorescence of nicotinamide and flavin adenine dinucleotides (NADH and FAD, respectively) can be detected without the aid of exogenous dyes (26, 29). Based on the autofluorescence of NADH and FAD, the optical reduction-oxidation (or redox) state of cells can be quantified as FAD/(NADH+FAD). This optical redox ratio (ORR) has been shown to be significantly correlated with mass spectrometry-based measurements of NAD^+^/(NAD^+^ + NADH), and can thus reveal the specific metabolic pathways engaged within a cell (30). Specifically, an increase in ORR has been attributed to increased oxidative phosphorylation because of the oxidation of NADH to non-fluorescent NAD^+^ and FADH_2_ to fluorescent FAD. On the other hand, hypoxia-like conditions that drive a buildup of NADH due to the inability to convert to NAD^+^ and increased glucose catabolism has been shown to decrease the ORR (30, 31). Recent work from separate groups has demonstrated that the optical redox ratio is sensitive to dynamic changes in oxygen consumption and can provide metabolic assessments comparable to those of the Seahorse metabolic flux analyzer (32, 33). The optical redox ratio has been used to create metabolic image maps of key organs (34), such as the heart and brain, identify metabolic changes associated with cancer progression (35, 36), determine cellular response to therapy (37–39), and discover a relationship between metastatic potential and cellular metabolism (32, 40, 41).

**Figure 1:**
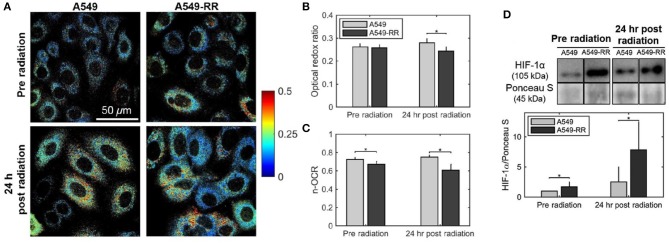
Optical redox ratio is sensitive to radiation-induced changes in cellular metabolism. Radiation causes a decrease in the optical redox ratio after 24 h in the radiation-resistant cells, indicating increased glycolytic metabolism. **(A)** Representative redox images of parental and radiation resistant A549 cells at baseline prior to radiation and 24 h after 2 Gy of radiation. **(B)** Quantification of redox ratio images indicates a statistically significant decrease in the optical redox ratio 24 h after radiation in the A549-RR cells compared with the parental A549 cells (*p* = 0.01). **(C)** Differences in the n-OCR (calculated as OCR/PPR) are consistent with the optical redox ratio. PPR refers to the proton production rate, which is equivalent to the extracellular acidification rate (ECAR). **(D)** Radiation causes a significant increase in HIF-1 in the radiation-resistant cells 24 h after radiation. Western blots of HIF-1 protein expression demonstrate statistically significant differences between A549 and A549-RR cells at baseline and 24 h after radiation, indicating reoxygenation-induced HIF-1 expression in the A549-RR cells. Asterisks placed above bars indicate statistical significance. Error bars in **(B,C)**, and **(D)** represent standard deviation of the mean plate value. Adapted with permission from Alhallak et al. (38).

Alhallak et al. determined the early metabolic alterations in response to radiation in human A549 lung cancer cells and an isogenic radiation-resistant clone (38). This clone was obtained by repeated exposure of parental radiation-sensitive human lung cancer cell line (A549) to ionizing radiation (25 fractions of 2.2 Gy every 3 days). Although there was no significant difference in ORR of radiation-resistant and -sensitive cells prior to administration of radiation, there was a significant decrease in ORR of radiation-resistant cells 24 h after radiation, which was consistent with Seahorse-based quantification of the normalized oxygen consumption rate (n-OCR) (Figure 1). The observed results indicate that the radiation-resistant cancer cells have decreased levels of oxygen consumption both at baseline and post-radiation and resort to increased glucose catabolism after radiation to potentially reduce ROS-induced toxicity. Interestingly, this radiation-induced decrease in the optical redox ratio was also associated with a large increase in the HIF-1 expression in the radiation-resistant A549 clone.

**Figure 2:**
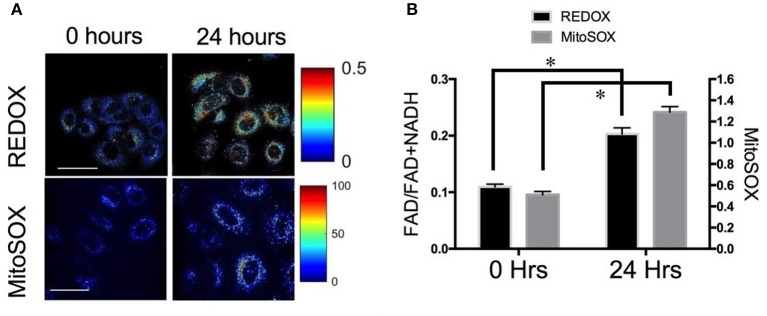
Optical redox ratio (ORR) is sensitive to changes in reactive oxygen species (ROS) **(A)** Representative images and **(B)** quantification of redox ratio and MitoSOX, a fluorescent reporter of mitochondrial ROS illustrates significant differences before and 24 h after treatment with YC-1. *p < 0.05. Adapted with permission from Lee et al. (39).

A subsequent by Lee et al. investigated metabolic changes in response to HIF-1 inhibition to determine if the changes in optical redox ratio post-radiation were indeed mediated by HIF-1 and a mechanism to avoid ROS-induced toxicity (39). They used multiphoton microscopy to determine the ORR of A549-RR prior to and post-treatment with YC-1, an established HIF-1 inhibitor. Treatment with YC-1 for 24 h resulted in a significant increase in the ORR compared with baseline, with a concomitant increase in mitochondrial ROS (Figure 2), a decrease in reduced glutathione and a decrease in glucose uptake (39). These results support the conclusion also reached by Furdui and colleagues who found increased glucose uptake that was utilized within the pentose phosphate pathway (PPP) to maintain the NADPH-glutathione buffer. This buffer helps scavenge radiation-induced ROS and hence promote radiation resistance (23). These results demonstrate the enormous potential of autofluorescence microscopy to not only provide clinically translational biomarkers of cellular response to therapy but also create opportunities for investigating radiation biology in live cells and animals at very high resolution.

### Lifetime Imaging

Fluorescent lifetime imaging microscopy (FLIM) measures the average time that a molecule spends in an excited state prior to emission. One significant advantage of FLIM over measurements of endogenous autofluorescence is that lifetime is independent of the fluorophore concentration. The lifetime of fluorophores, such as NADH and FAD depend on whether they are free or bound to a protein complex. For instance, the lifetime of NADH autofluorescence is shorter (~0.4 ns) when free and longer (~1 ns) when bound to protein complexes, such as malate dehydrogenase and lactate dehydrogenase while the lifetime of FAD autofluorescence is longer when free and shorter when bound to protein complexes, such as alpha-lipoamide dehydrogenase (42–46). By quantifying the ratio of free to protein-bound NADH and their respective lifetimes, FLIM can be used to identify the metabolic state of cells and tissue (42, 47–49). A recent study investigated the application of FLIM in radiation research (50). Campos et al. first treated human cancer cells and normal oral keratinocytes (NOK) with 10 Gy of radiation and recorded the resultant metabolic changes using FLIM. As early as 30 min post treatment, there was a significant decrease in NADH lifetime of cancer cells while there was no change in NADH lifetime of the NOK cells.

### Mitochondrial Organization

**Figure 3:**
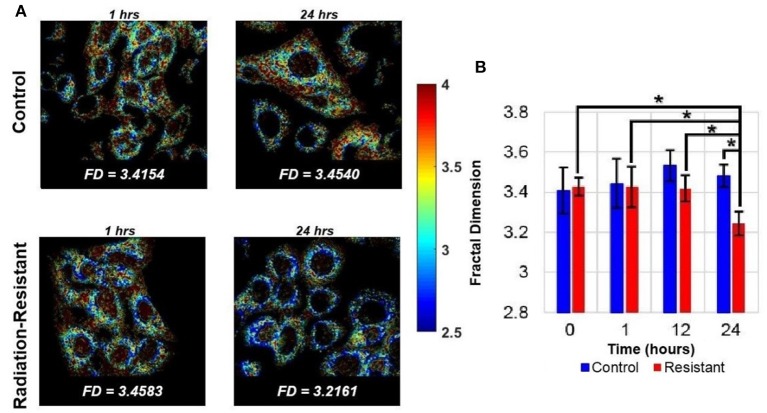
Quantifying mitochondrial organization from NADH autofluorescence. **(A)** Representative FD maps corresponding to lung cancer cells of both control and radiation-resistant groups. The images were obtained at time periods of 1- and 24-h post radiation. **(B)** Summary data demonstrates significant temporal changes in the mitochondrial organization of radiation resistant cell line at 24 h [Reproduced with permission from Vargas et al. (64)]. *p < 0.0001.

In addition to being the powerhouse of the cell, mitochondria are also critical to cell death pathways. The energy demands of a cell are maintained by a delicate balance between the rate of oxidative phosphorylation, tricarboxylic acid (TCA) cycle activity, structural changes to the mitochondrial network, and mitochondrial biogenesis. Mitochondria are continuously changing their organization through fission and fusion allowing for adaptation to different functional demands (51, 52). This dynamic mitochondrial network is sensitive to cell differentiation as well as oxygen and nutrient availability (30, 53–55). Fission is critical for mitochondrial biogenesis, cell division, and mitochondrial autophagy and manifests as numerous mitochondrial fragments. Fusion helps to maintain functionality through the sharing of proteins, genetic material, and metabolites and leads to the generation of interconnected mitochondria (56). Alterations to fusion-fission dynamics and hence the mitochondrial organization have been shown to be associated with several pathological conditions, including hypoxia-reoxygenation injury (57–59). Hypoxia-reoxygenation has been shown to result in a decrease in mitochondrial fusion and subsequent changes in length and shape of mitochondria (60). Targeting the changes in mitochondrial fusion and fission has been shown to protect cells from the effects of hypoxia-reoxygenation injury (61, 62). These studies of changes to mitochondrial structure in response to hypoxia-reoxygenation injury are highly relevant to radiation therapy due to the similarity in mechanisms generating oxidative stress. Radiation therapy leads to reoxygenation of previously hypoxic cells, thereby triggering a large production of mitochondrial ROS. The NADH autofluorescence images, which are used to calculate the optical redox ratio, can also be used to evaluate mitochondrial organization and specifically, fission and fusion. Specifically, Fourier-based power spectral density analysis of NADH autofluorescence images has been used to compute a metric termed mitochondrial clustering to quantify mitochondrial organization (30, 63). An increase in mitochondrial clustering was found during periods of increased glucose catabolism, such as hypoxia, resulting in more fragmented, or fissioned mitochondria. On the other hand, glutaminolysis was found to be associated with a decrease in mitochondrial clustering or more networked mitochondria (fusion). The same method was used to investigate mitochondrial structural dynamics in human skin *in vivo* (53). A recent study used an improved image processing method in the spatial domain to rapidly quantify the local fractal dimension (FD) within individual cells in response to radiation therapy (64). This analysis found a significant decrease in FD (or an increase in mitochondrial clustering) of radiation-resistant lung cancer cells between 12- and 24-h post-radiation compared with pre-radiation measurements. There were no significant changes in the radiation-sensitive cell population in response to radiation at any time point (Figure 3). The increased mitochondrial clustering observed here is consistent with the decreased optical redox ratio and increased glucose catabolism observed by Alhallak et al. using the same cancer cells (Figure 1) (38, 39).

## Optical Spectroscopy

Optical spectroscopy is a fiber-based approach using non-ionizing radiation to non-destructively and non-invasively examine tissue of interest. Their low cost and small footprint make “optical spectroscopy methods” an excellent tool for conducting pilot studies in animal models of cancer and in humans. Since optical measurements using the fiber optic probe are non-invasive or minimally invasive (depending on the tissue site), the same subject can be monitored multiple times a day or over weeks to evaluate response to treatment. In addition to its obvious benefits as a clinical adjunct to existing clinical imaging modalities that cannot be used every day on patients, optical spectroscopy obviates the need for sacrificing large numbers of animals at several time points in longitudinal studies to evaluate treatment response. Here, we describe two specific techniques—diffuse reflectance and Raman spectroscopy—that have demonstrated potential for monitoring radiation response in tumors and studying the differences between resistant and sensitive tumors.

### Diffuse Reflectance Spectroscopy

**Figure 4:**
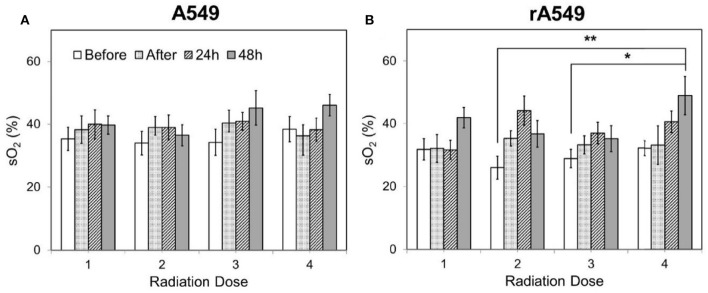
Changes in vascular oxygen saturation across all four doses of radiation for radiation-sensitive **(A)** and resistant tumors **(B)**. Each radiation dose presents the mean sO_2_ value for the following time points: 0 h, immediately after, 24 and 48 h post radiation. Data shown as mean ± SEM. *indicates *p* < 0.05 and **p < 0.01. Adapted with permission from Diaz et al. (84).

Diffuse reflectance or elastic scattering spectroscopy is an optical fiber- based technique for non-invasive interrogation of tissue. DRS uses optical fibers to deliver low-power non-ionizing light from a broad-band light source (400–650 nm) to tissue surface. The incident weak light undergoes multiple scattering and absorption events and is remitted back to the tissue surface as diffusely reflected light. Since the collected light has interacted non-destructively with the tissue, it provides a wealth of quantitative information about absorption and scattering, a combination of which is used for tissue pathology. Using models of light-tissue interaction that simulate the travel of photons within a scattering and absorbing medium, it is possible to quantify the diffusely reflected light and extract meaningful information related to tissue scattering as well as prominent tissue absorbers, such as oxygenated and deoxygenated hemoglobin (65–70). By exploiting the differences in light absorption spectra of oxygenated and deoxygenated hemoglobin, we can quantify the vascular oxygen content in tissue and obtain volume-averaged estimates of hemoglobin concentration. Measurements of vascular oxygenation have been shown to be concordant with microelectrode-based determinations of tissue oxygenation (71, 72) and immunohistochemical measurements of tumor hypoxia (73). Cell nuclei, mitochondria, and collagen are among the major contributors to light scattering in tissue and are known to undergo significant changes during disease progression (74). Taking advantage of these non-invasive and quantitative measurements, DRS has been used in several studies, with an eye toward clinical translation, for early cancer detection (75–77), prediction of response to therapy (78–80), and evaluation of tumor surgical margins (81). Given the importance of tumor oxygenation in radiation therapy, DRS can provide a non-invasive approach to quantify the biological response to radiation. Vishwanath et al. used DRS to longitudinally monitor tumor oxygenation and determine whether vascular oxygenation can identify treatment outcome earlier than tumor growth assays in a murine model of head and neck cancer treated with single dose of 39 Gy radiation. As early as 5 days post-radiation, radiation-responsive tumors exhibited faster and greater increase in vascular oxygenation compared with non-responding tumors (82). A more recent study from the same group found similar large increases in vascular oxygenation in both locally controlled and locally recurring tumors when the radiation dosage was split into five daily doses instead of a single dose. Additionally, the study also found that within the locally recurring group of tumors, a faster increase in reoxygenation during therapy was negatively correlated with recurrence time (83). Diaz et al. recently used DRS to study short-term changes in vascular oxygen saturation and hemoglobin concentration in radiation-sensitive and resistant A549 tumors treated with 4 dose fractions of 2 Gy (84), and also found significantly higher reoxygenation in radiation-resistant tumors 24 and 48 h after treatment (Figure 4).

This study was the first to report changes in reoxygenation kinetics measured in tumors which were established from a matched model of radiation-resistance. A matched model of radiation-resistance allows direct comparison of resistance-related features due to similar genetic background. While further studies are necessary to fully understand the mechanism of reoxygenation in the radiation-resistant tumors, results from other studies conducted using the same matched model of radiation resistance hint at the possibility of reduced oxygen consumption as a possible reason for the appearance of increased vascular oxygenation within the radiation-resistant tumors. Although the studies by Hu et al. (83) and Diaz et al. (84) used different cell lines in formation of tumor xenografts and treated them with different doses of radiation, they both showed that radiation-resistant tumors reoxygenate in response to radiation. These results are in agreement with a clinical study by Dietz et al. that used oxygen-sensing microelectrodes to measure pO_2_ in the cervical lymph nodes of head and neck cancer patients and found that increased reoxygenation correlated with poor radiation response (85). This suggests that DRS is a sensitive detector of reoxygenation and can provide valuable information about radiation response.

### Raman Spectroscopy

Raman spectroscopy offers the ability to probe biomolecular changes and visualize the complex molecular heterogeneity directly from cells and tissues (86, 87). Spontaneous Raman spectroscopy relies on the inelastic scattering of light, arising from its interactions with the biological specimen, to quantify the unique vibrational modes of molecules within its native context (88, 89). This exquisite chemical specificity of Raman spectroscopy has been exploited primarily within the realm of early detection of cancers of the oral cavity (90, 91), breast (92–98), cervix (99–101), and the brain (88, 102).

Recent studies have shown the presence of radiation-induced alterations in Raman spectral features and biochemical changes in cell lines with varying radiosensitivity (103, 104). The radiation response of single living cells has been studied to demonstrate dose-dependent changes in spectral features using principle component analysis (105, 106). In a series of human cancer cell lines treated with clinically relevant doses of radiation (<10 Gy), Matthews et al. found radiation-induced accumulation of intracellular glycogen in relatively radiation-resistant breast and lung cancer cell lines (107). Recent Raman spectroscopic studies on *ex vivo* lung and breast tumor xenografts have also identified elevated levels of glycogen in tumors exposed to a single, high radiation dose of 15 Gy (108, 109). These findings are of interest because separate non-imaging studies have identified a critical role for glycogen synthase kinase (GSK-3β) in the development of radiation resistance (110).

**Figure 5:**
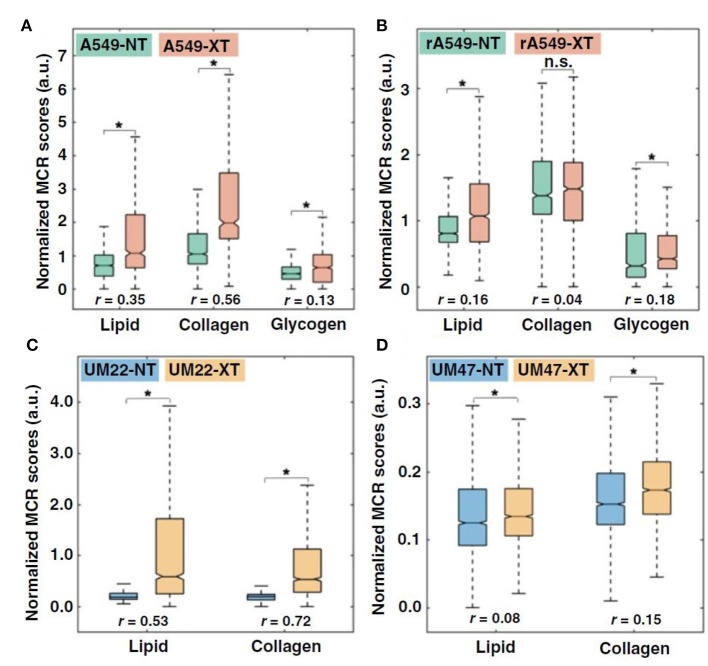
Quantitative MCR-ALS analysis of Raman spectra. **(A,B)**, Boxplots of normalized scores of lipid-rich, collagen-rich, and glycogen-rich MCR-ALS loadings showing radiation-induced differences in sensitive lung tumors (A549-NT vs. A549-XT) (NT: not treated, XT: X-ray treated) **(A)** and radiation-induced differences in resistant lung tumors (rA549-NT vs. rA549-XT) **(B)**. **(C,D)**, Boxplots of normalized scores of lipid-rich and collagen-rich MCR-ALS loadings showing radiation-induced differences in sensitive head and neck tumors (UM-SCC-22B-NT vs. UM-SCC-22B-XT) **(C)** and radiation-induced differences in resistant head and neck tumors (UM-SCC-47-NT vs. UM-SCC-47-XT) **(D)**. The effect size (*r*), characterizing magnitude of differences between groups, is provided for each comparison. *p < 0.001. Adapted with permission from Paidi et al. (112).

Radiation-induced changes in Raman spectra of excised cervical tumors have been shown to differentiate radiation responders from non-responders while pretreatment Raman spectra were incapable of predicting radiation response (111). In a recent study, Paidi et al. investigated whether radiation induced biomolecular changes detected by Raman spectroscopy could differentiate between radiation-resistant and sensitive tumors (112). They treated radiation-resistant and sensitive human head and neck (HN) and lung tumor xenografts with 2 Gy of radiation twice weekly for 2 weeks and conducted chemometric analysis using multivariate curve resolution-alternating least squares (MCR-ALS) to uncover biomolecular changes in the tumor microenvironment. MCR-ALS recovers the pure spectral profiles of the chemical constituents of the tissue specimen without a priori information of the composition of the specimen (113). Paidi et al. found an increase in lipid, collagen, and glycogen (lung only) levels for both sensitive and resistant lung and head neck tumors that were treated with radiation, with a much larger increase in the lipid-rich and collagen-rich signatures in the radiation-sensitive tumors (Figure 5) (112). Comparison of the treated tumors alone (RS-XT vs. RR-XT) pointed to a significantly higher collagen content in the sensitive tumors compared to their resistant counterparts in both lung and HN models, which could be attributed to radiation-induced fibrosis (114, 115). The lipid results are intriguing due to other studies that have found elevated levels of fatty acid synthase (FASN) in radiation-resistant cells (23). These findings demonstrate that clear spectral distinctions exist between radiation-resistant and sensitive tumors, and that these distinctions are consistent with recent work seeking to uncover the molecular mechanisms of radiation resistance.

## Discussion and Future Direction

The use of optical microscopy and diffuse optical spectroscopy presents exciting avenues for exploring radiation-induced changes across different length scales in cells and tissue. The technologies discussed in this review paper (summarized in Table 1)—although limited to superficial layers—are sensitive to two key hallmarks of tumors that play a critical role in radiation resistance—tumor hypoxia and metabolic reprogramming. While two-photon excited fluorescence from NADH and FAD can provide valuable information about specific metabolic pathways preferred by cells in response to radiation and the effect of such preferences on radiation resistance, Raman spectroscopy (or microscopy) can shed light on hitherto unknown biomolecular species in the tumor microenvironment that play a role in radiation resistance. Such studies have the potential to lead to new technologies centered on specific biomarkers for continuous monitoring during radiation treatment. Additionally, these studies can lead to the identification of novel therapeutic targets that can be exploited to possibly reverse radiation resistance. While optical spectroscopy has been at the forefront of optical technologies attempting to break into the clinical workflow, more work is required to establish baseline optical endpoints and the accuracy and reproducibility of these measurements. In addition, it will be necessary to associate these changes with specific outcomes corresponding to treatment response or failure. Optical spectroscopy has faced challenges with clinical translation, with attempts at early detection of cancer, discrimination between benign and malignant cancer, and demarcation of surgical margins not acquiring enough traction. The principal concerns in these clinical workflows was the perception that optical spectroscopy could never replace pathology, which is currently standard-of-care for these clinical problems. A possible advantage of advancing optical spectroscopy for measuring tumor response to therapy is the complete lack of any imaging technology or treatment biopsies that currently evaluate treatment response during the treatment regimen. While other imaging modalities such as optoacoustic imaging (OAI) can measure tumor oxygenation (116, 117), they have not yet been used in the context of radiation resistance. If decisions to escalate or de-escalate treatment for exceptional treatment responders or non-responders are to be made based on endpoints provided by optical techniques, near-perfect identification of treatment response within the first 1–2 weeks will be necessary to effect meaningful change. Tromberg et al. have demonstrated the ability of optical spectroscopy to provide an early indicator of chemotherapy response in breast cancer (78–80). Recent work has also significantly advanced the translation of non-linear optical microscopy from a laboratory-only method to the clinic for imaging the skin (118). The ability to translate two-photon excited autofluorescence from NADH and FAD to clinically compatible technologies, such as fiber optic probes could allow simultaneous determination of cellular redox state and mitochondrial fractal dimension *in vivo*. When combined with other information from DRS and RS, such as vascular oxygenation and biomolecular content, optical techniques could provide a powerful addition to a clinical workflow that could greatly benefit patients by improving response rates and quality of life.

**Table 1:** Comparison of optical microscopy and spectroscopy techniques for investigating radiation biology.

**Technology**	**Source of contrast**	**Quantitative endpoints**	**Advantages**	**Limitations**
Diffuse reflectance spectroscopy	Absorption and elastic scattering	Vascular oxygenation, vessel diameter, absorber concentration, tissue scattering	Non-invasive, low cost, portable	Limited penetration depth (1–2 mm); volume-averaged information
Raman spectroscopy	Raman (in-elastic) scattering	Contributions of individual molecular species (tissue-dependent)	High biomolecular specificity	Complex data analysis to extract meaningful biological information; limited penetration depth (1–2 mm)
Non-linear optical microscopy	Autofluorescence from NADH and FAD	Cellular redox state and local fractal dimension	High resolution, minimal out-of-focus photodamage	High cost, limited portability for clinical applications
	Fluorescence lifetime	Cellular redox state and protein-binding of NADH and FAD (bound vs. free)	Independent of fluorophore concentration	

## Author Contributions

SD and NR wrote the manuscript.

### Conflict of Interest

The authors declare that the research was conducted in the absence of any commercial or financial relationships that could be construed as a potential conflict of interest.
